# Resting state fMRI connectivity analysis as a tool for detection of abnormalities in five different cognitive networks of the brain in Multiple Sclerosis patients

**DOI:** 10.15761/CCRR.1000S1001

**Published:** 2016-07-30

**Authors:** Siamak P. Nejad-Davarani, Michael Chopp, Scott Peltier, Lian Li, Esmaeil Davoodi-Bojd, Mei Lu, Hassan Bagher-Ebadian, John Budaj, David Gallagher, Yue Ding, David Hearshen, Quan Jiang, Mirela Cerghet

**Affiliations:** 1 Department of Neurology, Henry Ford Hospital, Detroit, MI, USA; 2 Biostatistics and Research Epidemiology, Henry Ford Hospital, Detroit, MI, USA; 3 Department of Radiology, Henry Ford Hospital, Detroit, MI, USA; 4 Department of Biomedical engineering, University of Michigan, Ann Arbor, MI, USA; 5 Department of Radiology, University of Michigan, Ann Arbor, MI, USA

**Keywords:** multiple sclerosis (MS), cognition, resting state fMRI, diffusion tensor imaging (DTI), thalamus volume, fractional anisotropy

## Abstract

**Objectives:**

Cognitive dysfunction is present in at least half of patients with Multiple Sclerosis. The purpose of this study was to examine functional connectivity abnormalities in patients with multiple sclerosis (MS) using resting state fMRI (rsfMRI).

**Methods:**

Conventional MRI, rsfMRI and diffusion tensor imaging (DTI) data was acquired from 10 patients with relapsing-remitting multiple sclerosis (RRMS) and 20 healthy controls. Cross-correlation of the resting state average signal among the voxels in each brain region of the five cognitive networks: default mode network (DMN), attention, verbal memory, memory, and visuospatial working memory network, was calculated. Voxelwise analyses were used to investigate fractional anisotropy (FA) of white matter tracts. The normalized gray matter (GM), white matter and thalamus volumes were calculated.

**Results:**

Compared to controls, significant deficit in MS patients at each of five networks, attention (p=0.026), DMN (p=0.004), verbal memory (p<0.001), memory (p=0.001), visuospatial working memory (p=0.003) was found. Significant reduction (p=0.034) in the normalized GM volume and asymmetry in thalamus volume (p=0.041) was detected in MS patients compared to controls.

**Conclusion:**

Wide spread of functional abnormalities are present within different cognitive networks in patients with RRMS, suggesting that DMN may not be sufficient for measurement of MS cognitive impairment. Larger and longitudinal studies should ascertain whether rsfMRI of cognitive networks and changes in GM and thalamus volume can be used as tools for assessment of cognition in clinical trials in MS.

## Introduction

Multiple Sclerosis (MS) is a demyelinating disease of the central nervous system which affects more than 2.5 million individuals worldwide and is one of the most common causes of neurological disability in young adults. Cognitive impairment is recognized in 40%-70% of patients with MS [1,2] affecting multiple domains of cognition including attention [3], memory, and speed of information processing [4–6]. Over 50% of the patients with MS are unemployed within 10 years of diagnosis; and this is often attributed to a decline in cognition [7]. Possible explanation for cognitive decline include disruption of white matter tracts connecting the associative cortical areas [8,9], atrophy of white matter, possible cortical lesion and thinning of the cortex [10], changes in cortical cerebral metabolism [11,12] and reduction in regional cerebral blood flow in the frontal lobe [13]. Memory decline in patients with MS may evolve steadily from the onset of the disease and is not correlated with standard magnetic resonance imaging (MRI) parameters of the disease or clinical disease activity (relapses) [14]. Moreover, cognitive impairment is seen not only in patients with disabling MS but also in milder forms, so called “benign” MS [15]. fMRI studies in MS have focused mainly on working memory, using a modified PASAT (Paced Auditory Serial Addition Test) or N-back test for mapping brain activity during the cognitive tasks [16–19] suggesting that there are different activation patterns in working memory in MS.

Resting State fMRI (rsfMRI) connectivity analysis is based on assessment of the temporal correlations between different regions of the brain which at rest is organized in multiple neuroanatomic networks [20]. Growing bodies of studies are exploring the use of rsfMRI techniques in examining possible functional disconnectivity effects and direct comparison of resting-state functional connectivity with structural connectivity in different neurological disorders [21–24]. The Default Mode Network (DMN) is the most studied network in neurological, psychological or psychiatric conditions such as schizophrenia [24,25], autism [26], Alzheimer’s disease [22,27–29], depression [30], post-traumatic stress disorder [31,32] and aging [33]. In MS, dysfunction of DMN was more pronounced in cognitively impaired than in cognitively preserved patients with MS and correlated with structural changes in Diffusion Tensor Imaging (DTI) [21]. Few studies have examined resting state connectivity in networks other than DMN. Studies of cognitive networks have used event-related fMRI for evaluation of development of the attention network in children and adults [34] and recently rsfMRI to investigate memory encoding and retrieval networks [35]. Only one recent study explored large-scale neuronal networks of the brain using rsfMRI and found that functional abnormalities are present within and between large-scale neuronal networks in patients with MS, compared with healthy controls [36].

Apart from functional connectivity, changes in the white matter tract in MS patients have also been studied as a measure of anatomical connectivity. In one study, DTI tractography and graph theory approaches were used to investigate the topological organization of the white matter networks in patients with MS and was compared with healthy controls [37]. In a longitudinal study, Hannoun et al. have reported that Tract Based Spatial Statistics (TBSS) [38] analysis of DTI metrics show significant alterations in RRMS patients during a 2 year time period [39].

In the present study, in addition to DMN, we expand exploration of the rsfMRI connectivity analysis to four other cognitive networks (attention, verbal memory, memory, and visuospatial working memory) in patients with RRMS and compared them with controls. Our starting hypothesis was that MS deficit in the resting state is not only seen in DMN but also in other networks due to specific disease related damage of white matter tracts that connect brain structures.

## Materials and methods

### Subjects

The study was approved by the Henry Ford Health System institutional review board and written informed consent was obtained from all subjects. Ten (10) subjects with RRMS, diagnosed according to McDonald criteria were enrolled from the MS clinic at Henry Ford Hospital. Brain MRIs were obtained for all MS patients and controls as described in detail below. Electronic medical records were reviewed and data were collected on diagnosis criteria, socio-demographic factors, neurological and cognitive symptoms, and disability scores.

Twenty (20) healthy subjects (10 male and 10 female) with no prior history of neurologic or psychiatric disorders were enrolled as controls and had similar MRI procedures as MS patients.

### MR imaging

MRI was performed on a GE 3.0-T whole-body magnet using an eight channel phase array head coil. Resting state functional MRI images were acquired using a gradient echo EPI sequence with an FOV of 22 × 22 cm on a 64 × 64 matrix (3.4375 × 3.4375 mm in-plane resolution), 34 slices/3.5 mm slice thickness with BW/px=7.8125 kHz/px, TR/TE=2000 ms/30 ms. 150 volumes (five minutes) were recorded while the subjects were lying quietly with eyes open inside the scanner and using ear plugs for minimizing the noise from the scanner. Keeping the eyes open eliminates the coherent activity in the occipital cortex and leads to more reliable results compared to the situation where the eyes are closed [40]. Respiration and cardiac data of the patient were recorded during the scan using a pneumatic belt and pulse-oximeter, respectively. Artifact removal from the fMRI images was performed using this data, prior to image analysis.

To segment the brain images, high resolution inversion pulse prepared spoiled GRASS (IRSPGR) three-dimensional images were acquired with FOV of 24 × 24 cm, pixel dimensions 0.94 × 0.94 × 1 mm, 142 slices/1 mm slice thickness., TR = 8.816 ms, TE = 3.496 ms.

To detect MS lesions, T2 FLAIR images were acquired with FOV of 24 × 24 cm, TR = 8500 ms, TI = 2550 ms, TE = 80 ms, imaging matrix 256 × 256 with pixel dimensions 0.94 × 0.94 mm^2^, 32 slices/4 mm slice thickness.

Also q-ball images were acquired with 55 diffusion gradient directions with b-value = 1500 s/mm^2^ and six B0 reference measurements were performed using spin echo diffusion-weighted 2D echo-planar imaging with FOV 24 cm, TR = 8s, TE 94ms, slice thickness of 2.6 mm, and in-plane pixel 2.5 × 2.5 mm^2^ and 96 × 96 imaging matrix interpolated to 0.975 × 0.975 mm^2^ and 256 × 256 matrix.

### Image processing

We previously reported a framework that we designed for studying changes in the functional connectivity network for stroke patients using resting state fMRI images [41]. We used the same framework for MS patients with the difference that for the stroke study, all possible regions in the brain were studied but in the current study, only regions related to the cognitive networks are considered; in the next few sections we will describe the details of this framework and the modifications employed for the MS study.

#### Segmentation of the anatomical images and definition of ROIs for cognitive networks

Our framework is based on extracting the average fMRI signal from segmented regions in the brain and using these signals for calculating the functional connectivity between these regions. Therefore one of the major steps of our analysis is segmenting the fMRI images. For this purpose, we used the 3D IRSPGR images as the reference for segments of the brain regions. Initially, the 3D images were skull stripped using the BET (Brain Extraction Tool) [42] module in FSL (FMRIB Software Library) [43]. We used the HAMMER package (University of Pennsylvania, Section of Biomedical Image Analysis, SIBA) for segmenting the skull stripped image [44]. This software produces labels for anatomical regions of the brain corresponding to gyral and subcortical brain structures, such as hippocampus, superior temporal gyrus, superior frontal gyrus, etc. Using this software, the 3D IRSPGR image of the brain was segmented into 104 regions; however, we used only 94 of these regions in our functional connectivity study, excluding segments such as the ventricles which have no functional role.

We investigated five circuits in the brain responsible for five primary cognitive functions. These circuits or networks are identified below:

### Default Mode Network (DMN)

The DMN was defined for the following brain regions [21,36]

(1) Medial Prefrontal Cortex (2) Rostral Anterior Cingulated Cortex (3) Posterior Cingulated Cortex (4) Precuneus (for the Posterior Parietal Cortex), and (5) Lateral Parietal Cortex

In our study, we considered the entire cingulated cortex as one region, and defined the DMN with these five regions.

### Attention [34,45]

(1) Thalamus (Superioir Colliculus and Pulvinar) (2) Superior Parietal Lobule (3) Middle Frontal Gyrus (4) Superior Frontal Gyrus (5) Angular Gyrus and (6) Supramarginal Gyrus

### Verbal memory [46–48]

(1) Inferior Temporal Gyrus (2) Medial Occipito-Temporal Gyrus (3) Middle Temporal Gyrus, (4) Temporal Pole (5) Angular Gyrus, and (6) Inferior Frontal Gyrus

### Memory [49–51]

(1) Angular Gyrus (2) Fusiform Gyrus (lateral Occipito-Temporal Gyrus) (3) Superior Parietal Lobule (4) Supramarginal Gyrus (5) Hippocampal Formation, and (6) Superior Frontal Gyrus

### Visuospatial working memory [49]

(1) Cingulate Region (2) Superior frontal Gyrus (3) Fornix (4) Superior Parietal Lobule (5), and Supra Marginal Gyrus (for Lateral Parietal or PWM)

**Figure 1:**
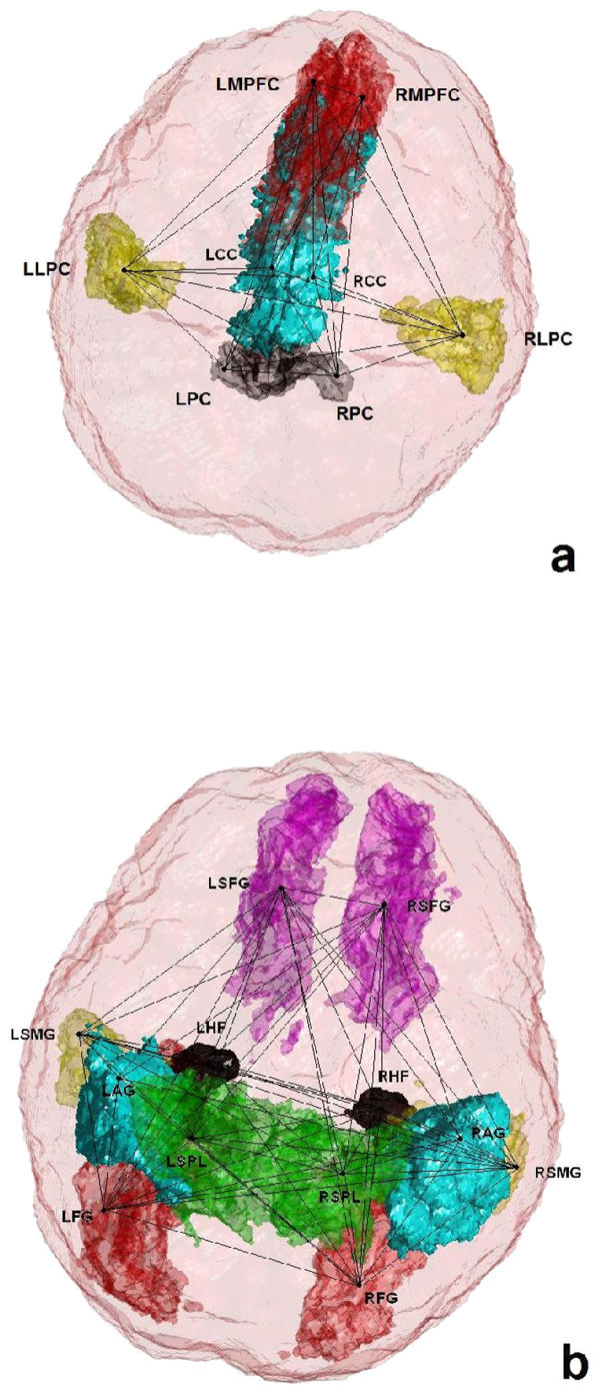
3D visualization of the regions involved in the (a) Default Mode Network (b) Memory Network (L, Left; R, Right; AG, Angular Gyrus; FG, Fusiform Gyrus; SPL, Superioir Parietal Lobule; SMG, Supramarginal Gyrus; HF, Hippocampal Formation; SFG, Superior frontal Gyrus; MPFC, Medial Prefrontal Cortex; CC, Cingulated Cortex; PC, Precuneus; LPC, Lateral Parietal Cortex).

Figure 1 shows three dimensional visualization of the default mode and memory networks. For each of the regions mentioned above, there exists one region on the left and one on the right hemisphere so the actual number of regions considered in our analysis is twice the number noted above. The total number of possible links for a network with *n* nodes is $\frac{n\left(n-1\right)}{2}$, therefore, for the five networks described above, there exist 28, 45, 66, 66 and 45 links respectively.

#### Image coregistration

To extract the fMRI signals from each of the corresponding segmented regions in the anatomical images, coregistration of these two images was necessary. The resolution and FOV of the IRSPGR image differs from the fMRI images. In addition, there is a high likelihood of subjects moving between the scans. Since the fMRI images are spatially distorted, unwarping was also a necessary step for coregistration. For coregistering the fMRI and ISRPGR images, the FLIRT [52] module of FSL was used. Affine transform with 12 degrees of freedom ensured warping of the images along with the other steps of coregistration. After coregistering this image to the fMRI images, the average fMRI temporal signal corresponding to all the voxels in each segmented region can be extracted. These are the signals used for connectivity analysis.

#### Resting state fMRI connectivity analysis

The preprocessing step of the resting state data was mainly based on the method suggested by Fox *et al.* [53] for time-slicing correction, and realignment for correcting the motion artifact and spatial smoothing using Statistical Parametric Mapping (SPM8) (www.fil.ion.ucl.ac.uk/spm) software. Next, having the recorded physiological data during acquisition of the fMRI images, cardiac and respiratory noise was filtered from the fMRI data using a linear regression model. And finally the temporal signals were low pass filtered to contain only signals with frequency components less than 0.08 Hz. At this stage, the fMRI images were ready for the functional connectivity analysis.

After finding the average signal among the voxels in each ROI described in the segmentation section, to measure the strength of functional connectivity between two different brain regions, cross correlation of these signals was calculated. Cross-correlation is a measure of similarity of two waveforms and in the context of brain waves; it shows the coherence of two signals originating from two different regions of the brain. By calculating the cross correlation between every two region pairs in the brain, the functional connectivity network matrix can be found.

For each subject in the control database and for the ten MS patients, the five cognitive networks were defined as described in the segmentation section. Cross correlation of the average fMRI signal between every possible pair of the regions within each of the five networks was calculated and for each network, the cross correlation matrix was created. Each matrix element in these matrices represents the connectivity strength between every two nodes that are being connected through that link. The mean and standard deviation of the elements of these matrices were calculated across the controls to be used as thresholds for finding the abnormal connectivity links of the cognitive networks in the MS patients. For every MS patient, the cross-correlation values of all the links in each cognitive network were compared against the same link among the normal controls of the same gender and all the links in the MS patients that had a value outside the range (Mean ± 2 SD) of the controls were marked as abnormal.

#### Effects of the MS lesions on the white matter tracts

One of the MR modalities that is used for detection of MS lesions in the white matter, is T2-Fluid-attenuated Inversion-Recovery (T2-FLAIR) MR imaging. In these images, except for necrotic lesions, MS lesions are seen as hyper-intense signals [54]. We used these images as guides to locate the lesions and to investigate how they correlate with the changes in the FA value in the white matter tract.

Our analysis method was based on preprocessing the FA images using the TBSS [38] module of FSL. TBSS enables voxelwise statistical analysis of FA images across subjects to investigate changes in the white matter tract. Using the q-ball diffusion images, FA maps were created for the normal control group and the MS patients. For mapping only the white matter in the FA images, a threshold of 0.2 was used. All the FA images (and the T2-FLAIR images) were nonlinearly co-registered to the standard FMRIB58_FA template which is a high resolution average of 58 well-aligned FA images from healthy male and female subjects and can be found in the TBSS package. After registration, the mean FA image of the normal controls was created and the white matter tract was skeletonized. Voxel-wise t-test was performed between the skeletonized FA maps of every MS subject and the FA maps of the controls to detect those voxels that have FA values which are significantly different compared to the control group. After evaluating the FA values in the MS subjects using this method, the maps were overlaid on the T2-FLAIR images to explore the effects of the MS lesions on the FA values of the white matter tracts.

#### Volumetric analysis of the brain structures

After segmentation of the brain regions using the 3D IRSPGR images, a volumetry study was done on all the segmented brain regions in controls and MS patients based on their normalized sizes. First the volumes of the segments were normalized to the total brain volume of each subject, to be able to compare the volume of these segments between the patients and controls. We divided the segmented regions into three groups of white matter (WM), gray matter (GM) and Cerebrospinal Fluid (CSF) (the lateral and third and fourth ventricles and subarachnoid CSF) and defined the total brain volume as the sum of all these segments. Next by adding the volumes of the segments in the gray and white matter groups, found the total normalized gray and white matter volumes in each subject.

Also, since we observed unilateral changes of the volume of the thalamus in the MS patients, therefore, to quantify the size change, we calculated the Asymmetry Index (AI) for the thalamus volume as:

$$AI=\frac{V_{LT}-V_{RT}}{\frac{V_{LT}+V_{RT}}{2}}$$

Here *V_LT_* and *V_RT_* are the normalized volumes of the left and right thalami.

### Statistical analysis

The data (total number of links to each network) were evaluated for normality. Data transformation would be considered if data were not normal. A one sample t-test was used to test MS links that significantly differed from the controls. The Pearson correlation coefficient was calculated between the five networks. Two-sample t-test was used to test difference of gray matter, white matter, and thalamus AI between MS and controls.

## Results

Ten patients with RRMS were studied and compared with healthy subjects. Socio-demographic data, disability scores (EDSS), duration of the disease and complaints of cognitive problems are summarized in Table 1.

### Resting state connectivity of analyzed networks

**Figure 2:**
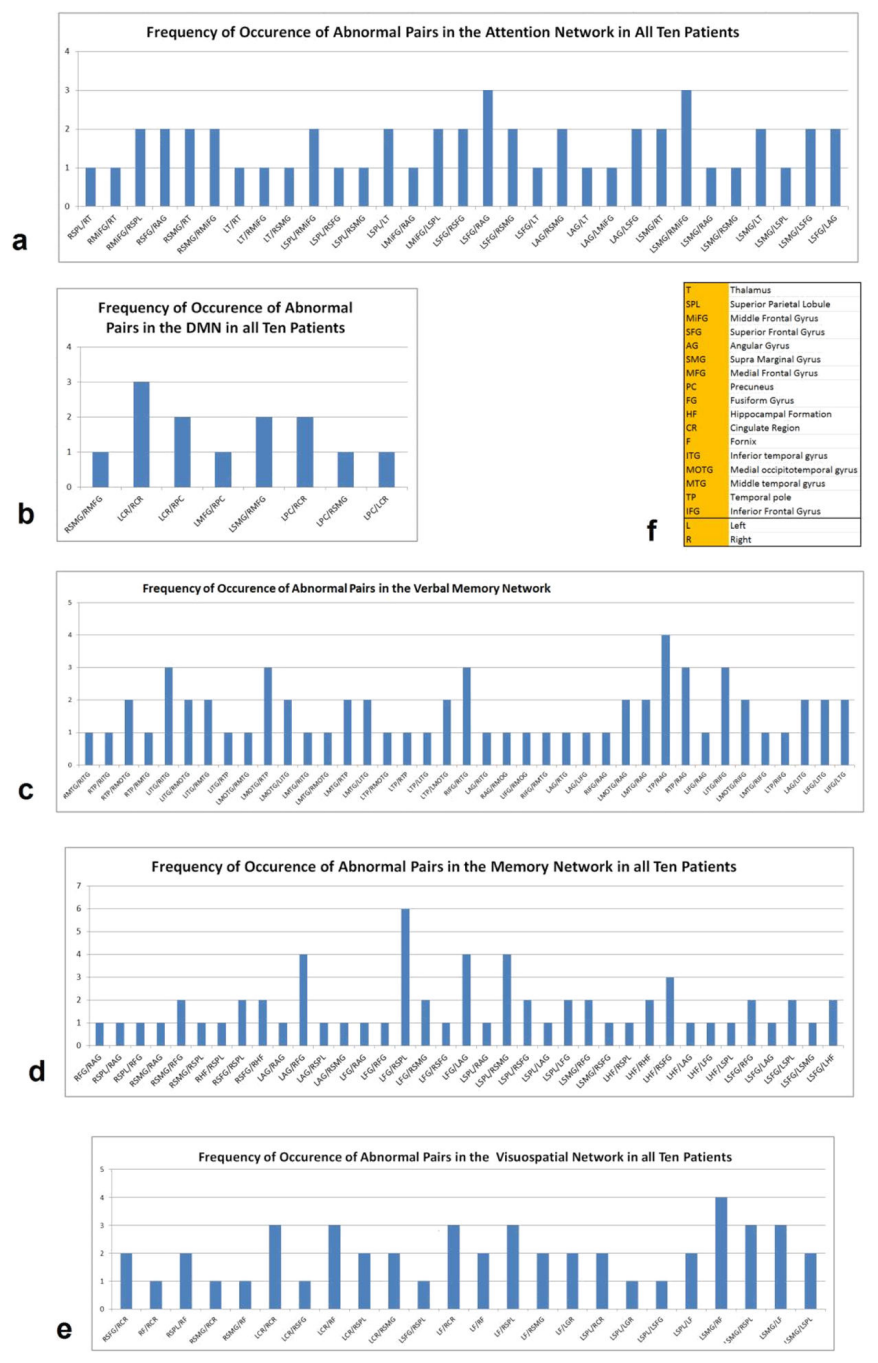
Frequency of occurrence of abnormal functional connectivity links in the (a) DMN (b) Verbal Memory (c) Attention (d) Memory (e) Visuospatial Working Memory networks and (f) the abbreviations used in these graphs.

To find the links or regions that were most affected among the group of MS patients, we summed all the abnormal links in each cognitive network across all the MS patients. Figure 2 summarizes the frequency of occurrence of abnormal links in the five networks studied. In the DMN, MS patients had decreased correlation between right and left cingulate regions, and left and right precuneus and the left supramarginal gyrus and the right medial frontal gyrus. In the attention network, decreased correlation between left superior frontal gyrus and right angular gyrus and left supra marginal gyrus and right middle frontal gyrus were recorded in MS patients. The verbal memory network analysis of MS patients demonstrated decreased correlation between right and left temporal structures. In memory network analysis, decreased correlation between left fusiform gyrus and right superior parietal lobule was observed while in the visuospatial working memory network there was decreased correlation between left supra marginal gyrus and right fornix among other links related to the left and right fornix.

Significantly reduced connectivity (decreased correlation) of cognitive networks in MS patients was detected in attention (p=0.026), DMN (p=0.004), verbal memory (p<0.001), memory (p=0.001) and visuospatial working memory (p=0.003) networks compared with controls (Table 2). The DMN was highly correlated with attention (r=0.76, p=0.011), verbal memory (r=0.69, p<0.026), and visuospatial working memory (r=0.69, p=0.028) networks, but did not significantly correlate with the memory network (r=0.42, p=0.221), indicating that DMN may not be sufficient measurement for MS cognitive impairment. In addition, significant correlations were also detected between attention and verbal memory (r=0.67, p=0.034), attention and visuospatial working memory (r=0.85, p=0.002) networks, respectively.

### MRI structural analysis

To evaluate the structural damage related to network connectivity systems we assessed several anatomical parameters: brain volume, gray/white matter volume, thalamus volume, and white matter tracts (DTI measurements). Table 3 lists the values for the GM volume, WM volume and thalamus AI in the MS patients and the controls. Analysis of the normalized volume of the GM showed significant reduction (p=0.034) in the MS patients.

Also, we observed considerable change in the size of the thalamus in MS patients in either the left or right thalamus. The thalamus AI for the MS patients (0.188 ± 0.172) is considerably higher (p=0.041) than the normal controls (0.089 ± 0.057).

**Figure 3:**
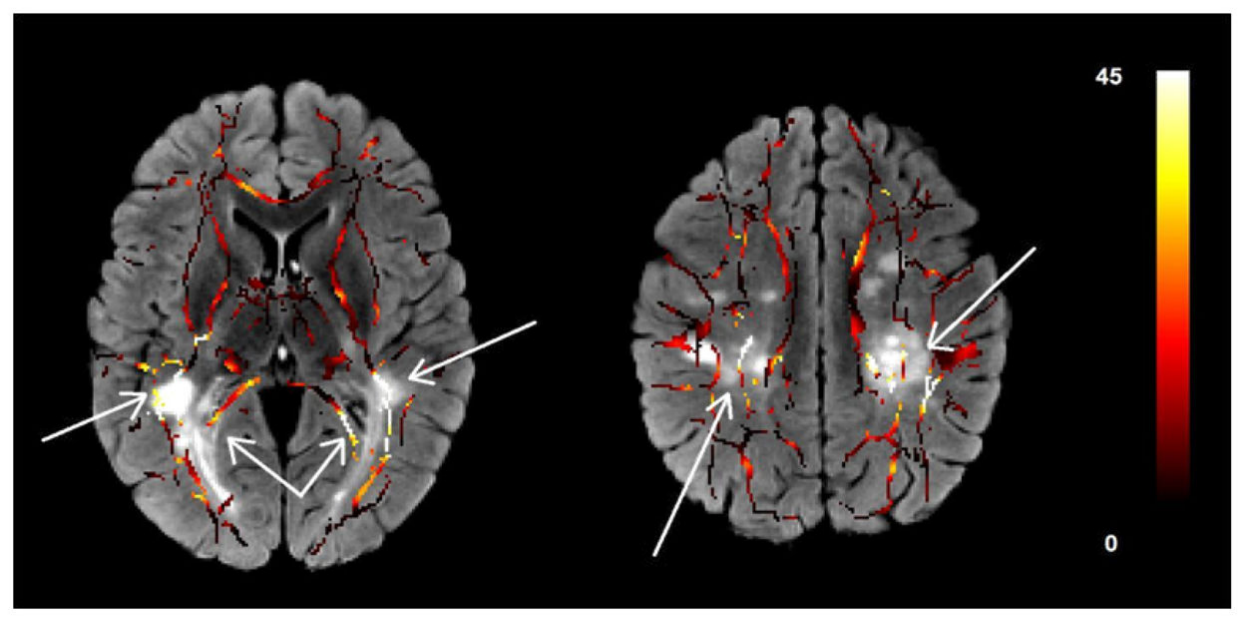
Two slices of the skeletonized white matter tracts overlaid on the T2-FLAIR images. The color of the white matter tracts shows the difference of the FA value in that voxel with the same voxel in the normal control group after registration to the target ATLAS. These images show the anatomic relation between the reduction of the FA values and the existence of the MS lesions as seen in the T2-FLAIR maps.

Figure 3 shows two slices of the skeletonized white matter tract overlaid on the T2-FLAIR image of the same patient. The MS lesions on the T2-FLAIR map are visualized as bright patches. The color of the skeletonized white matter tract is an indicator of the voxelwise difference of the FA map of the MS patient from the normal controls. We found the largest difference in the FA value in MS patients when compared with the same segments in the controls in the areas around the lesions in the T2-FLAIR image. The FA values in the areas far from the lesions do not show significant difference with the controls. These patterns can be seen in most of the white matter tracts in these subjects.

## Discussion

We analyzed functional connectivity of the five cognitive networks (Default Mode Network attention, verbal memory, memory and visuospatial working memory) using resting state fMRI in patients with RRMS and compared with healthy subjects. Significant decreases in functional connectivity in MS patients were not only detected in DMN but also in four other cognitive networks. MS patients exhibited reduced normalized grey matter volume and increased AI of thalamus volume. In addition, DTI data detected abnormality of anatomic white matter tracks which affect functional connectivity. Our results demonstrate that in MS patients, MRI measurements could be very useful in detecting abnormalities in functional and anatomic connectivity of brain cognitive networks other than DMN.

In our study, the MS patients had various and significant levels of weakening of the links between the nodes in these five brain networks. By studying the frequency of occurrence of these abnormal links in each network across all patients, we observed that certain links in each network have been affected more than others within each patient. This variability of the links affected in MS patients may be due to heterogeneity of the disease, not only in clinical symptoms, clinical course and response to treatment but also in involvement of neuroanatomical structures, commonly seen between patients. Few regions contribute to more than one cognitive network studied, however, the high correlation between the DMN and the attention, verbal memory and visuospatial working memory networks suggest that although multiple sclerosis affects more than one cognitive network, there may be common pathways or relays for these networks. Our results of the DMN analysis agree with a recent report in literature [21] of decreased correlation in cingulated region and lateral parietal cortices in MS patients, which was suggested to be responsible for accumulation of cognitive deficit.

In our study we did not find a correlation between reported cognitive impairment and abnormalities in functional connectivity. It is possible that patient’s perception about their cognitive state does not reflect their true functioning. Another reason could be the small cohort of patients studied. Not having a formal neuropsychological testing of our MS patients at the time of MRI study was a limitation. Two of our patients had formal, complete neuropsychological evaluation. As seen in Table 4 (supplemental data) different degrees of impairment were found in many cognitive functions: processing speed, attention, working memory, verbal memory and visuospatial recall. Although clinical assessment of the cognitive function of the two MS patients shows correlation with the rsfMRI connectivity results, considering the small sample size, drawing a conclusion on the correlation of the rsfMRI connectivity findings and the cognitive deficit state would be difficult. However, our results suggest that rsfMRI can be a promising method for assessing and quantifying the level of cognitive deficit in MS, and other neurological and psychiatric disorders and studying a larger patient database may derives a solid conclusion about the correlation of the connectivity analysis results and the level of cognitive deficit.

Cognitive impairment and disruption of DMN were correlated with grey matter abnormalities in MS. Previous studies showed that brain atrophy was a predictor of cognitive impairment [55,56] and thalamic lesions affected DMN and contributed to cognitive dysfunction [57]. In line with these findings, one of the major biomarkers in our study that showed significant difference between the MS patients and the controls is the normalized global GM volume (p=0.034). Also, volumetric analysis of the thalamus in normal controls and MS patients in our study showed association of large volume changes of this region with the disease. Compared to the controls, there is a significant increase in the value of the thalamus AI in the MS patients. The association between thalamic atrophy, altered functional connectivity and clinical and cognitive dysfunction in patients with MS were previously analyzed using resting-state magneto encephalography (MEG) [56]. However, our data did not detect significant differences in total thalamus volume between MS patients and the controls (p=0.66). Instead, in our study, the thalamic AI value exhibited significant difference (p=0.041) between MS patients and the controls, which indicate that thalamic AI may be a sensitive biomarker for MS patients; this requires further investigation. Contradictory findings in thalamic volume and cognitive impairment are found in literature. In some studies, changes in the FA values of the thalamus and the size of the thalamus were linked to cognitive performance of MS [57–59] while in another study, thalamic volume reduction did not differ between cognitively impaired and cognitively intact MS patients [58]. However, all these reports confirm our findings related to the changes in the thalamus seen in MS patients.

Furthermore, our results show that presence of lesions in the T2-FLAIR maps correlates with reduction in the FA value in the white matter tracts adjacent to these networks’ brain centers. This suggests weakening of the anatomical connectivity between the areas that these tracts are connecting. These results agree with reports by Rocca *et al.* [21] where abnormalities in corpus callosum and cingulum in MS patients were found using diffusion tensor imaging. In their study the results of neuropsychological tests and diffusion tensor MRI abnormalities correlated in these two regions. In another study, Mesaros *et al.* [59] correlated cognitive impairment in MS with global and local changes in DT tractography; however, neither of these studies evaluated WM tracts belonging to the specific cognitive networks. Brain networks have been recently studied by Shu *et al.* [37] using diffusion tensor tractography and graph theory methods to show that brain network topological efficiency in MS patients is reduced compared to controls. Their focus, however, was on sensorimotor, visual, default-mode and language systems. Indeed combining analysis of resting state connectivity and diffusion tensor imaging was found to show significant agreement and provide an improved representation of neuronal connectivity in both healthy and pathological states [60]. One direction to extend our study is to isolate the WM tracts that directly and indirectly connect the brain regions in each of these cognitive networks and to investigate the correlation of the rsfMRI connectivity values with the weakening of the links in these networks.

## Conclusions

In conclusion, in our preliminary study, decrease in functional connectivity was present within different cognitive networks in patients with RRMS along with asymmetry of the thalamus volume and atrophy of the cortical areas. This confirms that presence of cognitive impairment is common in MS. Larger and longitudinal studies are needed to confirm our results and ascertain whether rsfMRI of cognitive networks and changes in GM and thalamus volume can be used as non-invasive evaluations of cognition in clinical trials in MS. In addition, voxelwise statistical analysis of FA data appeared promising for detecting abnormality in anatomic connectivity. Further study is warranted to investigate the relationship between cognitive brain circuits and both functional and anatomic connectivity.

## Figures and Tables

**Table 1:** Socio-demographic and clinical characteristics of subjects.

	RRMS (N =10)	Controls (N = 20)
**Gender: Female/Male**	9/1	10/10
**Race: White/Black/Other**	7/3/0	11/0/9
**Mean Age (Range)**	37.6 (20–54)	33.3 (18–58)
**Median Disease Duration (Range)**	7 (5–20)	
**Mean EDSS (Range)**	3.2 (1–7.5)	
**Cognitive Complaints: Yes/No**	6/4	

**Table 2:** Cognitive deficit in the MS patients at each the of five networks (std, standard deviation).

Cognitive Network	Reduced Connectivity; Mean (std)	p-Value
**DMN**	2.800 (±2.347)	0.004
**Attention**	3.000 (±3.559)	0.026
**Verbal Memory**	3.000 (±2.828)	<0.001
**Memory**	3.400 (±2.3190)	0.001
**Visuospatial Working Memory**	3.200 (±2.5733)	0.003

**Table 3:** Normalized Gray Matter (GM) volume, White Matter (WM) volume and Thalamus Asymmetry Index values in the patients and controls (GM, Grey Matter; WM, White Matter; std, standard deviation).

Normalized Volume	RRMS (N=10); Mean (std)	Controls (N=20); Mean (std)	p-Value
GM volume	0.435 (±0.021)	0.453 (± 0.011)	0.034
WM volume	0.297 (±0.048)	0.281 (±0.0168)	0.1794
Thalamus asymmetry index	0.188 (±0.172)	0.0894 (±0.0572)	0.041

**Table 4:** Neuropsychological evaluation of patients P2 and P6.

	P6	P2
**Processing Speed**	borderline impaired	impaired
**Attention**	impaired	impaired
**Working Memory**	impaired	below average
**Visual Scanning**	low average	impaired
**Verbal Abstract Reasoning**	low average	below average
**Recall**	low average	impaired
**Learning**	low average	impaired
**Visual Recall**	Impaired	impaired
**Language**	low average-impaired	poor-impaired
**Visuo-Spatial Skills**	intact	poor-impaired
